# Overexpression of CISD1 Predicts Worse Survival in Hepatocarcinoma Patients

**DOI:** 10.1155/2022/7823191

**Published:** 2022-03-11

**Authors:** Tailiang Lu, Chenglong Li, Cailing Xiang, Yongqiang Gong, Wei Peng, Chaowu Chen

**Affiliations:** Department of General Surgery, Hunan Provincial People's Hospital (the First Affiliated Hospital of Hunan Normal University), Changsha, Hunan Province, China

## Abstract

**Background:**

Ferroptosis plays a vital role in hepatocellular carcinoma (HCC). CISD1 is known to regulate ferroptosis negatively. However, the correlations of CISD1 to prognosis in HCC and its potential mechanism remain unclear.

**Aim:**

To investigate the expression level and prognostic value of CISD1 in HCC.

**Methods:**

Gene expression and clinical data for 33 cancer types in TCGA were downloaded from the UCSC Xena platform. Pan-cancer analysis was performed to determine the expression profile and prognostic value of CISD1 in human cancers. GEO datasets and Human Protein Atlas (HPA) were used to verify the mRNA and protein expression levels. The influence of CISD1 on clinical prognosis in HCC was evaluated using a Kaplan-Meier plotter. The PPI network was constructed using the STRING database and Cytoscape. GO and KEGG pathways were constructed using the “clusterProfiler” R package with the FDR cutoff of 0.05. The methylation at the CISD1 promoter was detected using UALCAN and GEO datasets. The correlations between CISD1 and HCC immune infiltrates were investigated via TIMER.

**Results:**

Pan-cancer analysis of TCGA data showed that CISD1 is differentially expressed in multiple tumors. Data of gene expression microarrays reveal that the mRNA expression of CISD1 is higher in HCC than that in normal tissue. The protein level of CISD1, validated by the Human Protein Atlas (HPA) database, was upregulated consistently with mRNA levels in HCC samples. High CISD1 expression was associated with better overall survival (OS), disease-free survival (DFS), disease-specific survival (DSS), and progression-free survival (PFS) in LGG, but with poorer OS, DFS, DSS, and PFS in LIHC. Protein-protein interaction (PPI) analysis and GO/KEGG analysis showed that the PPI network and GO term of CISD1 were mainly associated with energy and iron metabolism. Promoter hypomethylation correlated with overexpression of CISD1. CISD1 expression was positively correlated with infiltrating levels of CD8+ T cells, macrophages, neutrophils, and dendritic cells (DCs) in HCC.

**Conclusions:**

These findings suggest that hypomethylation of the CISD1 promoter increases its expression in HCC. CISD1 is associated with prognosis and immune infiltrating levels of CD8+ T cells, macrophages, neutrophils, and DCs in HCC patients. These findings suggest that CISD1 can be used as a prognostic biomarker for determining prognosis in HCC.

## 1. Introduction

According to the World Health Organization's (WHO's) recent update, liver cancer is ranked third based on mortality worldwide after lung and colon cancers [1, 2]. Fifty percent of all liver cancer patients in the world are in China, posing a heavy burden on China's medical and health services [3, 4]. Although the incidence of HCC has declined, disease-specific mortality remains high [5]. Early diagnosis is vital to improving the prognosis of liver cancer patients. In the past 20 years, a large number of molecular biomarkers including microRNAs, protein-coding genes, long noncoding RNAs, and methylated gene promoters are abnormally expressed in HCC patients, and most of them have potential clinical application value [6]. However, the pathogenesis of HCC is complex. It involves cell cycle regulation and signal transduction and the interaction of multiple genes at multiple steps [7]. New drug targets may be discovered via screening networks of genes associated with tumor formation, progression, and metastasis.

Distinct from apoptosis, necrosis, and pyroptosis, ferroptosis is an oxidative, iron-dependent form of cell death. It is a form of regulated passive cell death that is closely associated with drug-resistant diseases [8]. Recent studies have shown that ferroptosis plays an important role in anticancer drugs resistance [9]. Artesunate resistance of head and neck cancer (HNC) cells is resulted due to the activation of the Nrf2–ARE pathway. The Nrf2 inhibition reverses the resistance of cisplatin-resistant HNC cells to artesunate-induced ferroptosis [10]. Inhibition of ferroptosis in gastric cancer contributes to its decreased sensitivity to paclitaxel and cisplatin leading to tumor growth [11], suggesting its significant translational effects in gastrointestinal tumors' treatment. Previous studies have reported that ferroptosis plays a vital role in HCC [12]. Sorafenib is the first-line therapeutic agent for liver cancer. It can block the angiogenesis and growth of liver cancer and significantly improve OS and TTP in patients with advanced liver cancer [13]. Nevertheless, some hepatoma cell lines are less sensitive to sorafenib-induced ferroptotic cell death. Recently, Wang et al. showed that upregulation of Glutathione S-transferase zeta 1 (GSTZ1) enzyme enhanced sorafenib-induced ferroptosis in HCC cells by inhibiting the NRF2/GPX4 axis [14]. CISD1, a CDGSH iron-sulfur domain-containing protein, has been localized in the outer membrane of the mitochondrion and is known to regulate ferroptosis negatively [15]. CISD1 is linked with cell oxidation processes and plays a key role in the regulation of cellular respiration and ferroptosis. It has been reported to suppress the activation of autophagy and contribute to breast cancer progression [16]. Recently, Li et al. reported that metaxin 1 (MTX1) upregulation in HCC contributed to sorafenib resistance possibly involving CISD1 mediated autophagy mechanisms [17].

Although levels of CISD1 mRNA are significantly increased in different human cancer cells [16, 18–21], the expression of CISD1 in HCC and its role are still not fully elucidated. Therefore, this study used bioinformatics analysis to explore the potential role and mechanisms of CISD1 in the context of HCC pathogenesis from various aspects and different levels.

## 2. Materials and Methods

### 2.1. Datasets and Different Gene Expression Analysis

Gene expression and survival data for 33 cancer types were acquired from TCGA TARGET GTEx cohort in the UCSC Xena platform [22]. mRNA expression data (GSE14520, GSE25097) and methylation data (GSE54503) of hepatocellular carcinoma were obtained from Gene Expression Omnibus (GEO) [23, 24]. Different expressions of CISD1 in pan-cancer were detected by the Wilcoxon rank-sum test via the R package. The ggplot2 package [25] was used to visualize the results. Protein expressions of CISD1 were collected from the Human Protein Atlas (HPA) website (https://www.proteinatlas.org/).

### 2.2. Survival Analysis

Survival analysis was performed using a univariate Cox regression hazard model, and survival curves were derived from the Kaplan-Meier survival analysis using the R package “survival” [26]. To confirm the prognostic value of CISD1 in hepatocellular carcinoma patients, the Kaplan-Meier plotter database was used employing the effect of 54,675 genes on the survival of 10,461 cancer samples. The correlation between CISD1 expression and survival in hepatocellular carcinoma was analyzed by the Kaplan-Meier plotter (http://kmplot.com/analysis/) [27].

### 2.3. Protein-Protein Interaction (PPI) Analysis

The String [28] database was used to create the interaction network of CISD1. A protein was considered interacting with CISD1 in the network if the interaction score was more than 0.7. The active interaction sources include text mining, experiments, databases, coexpression, neighborhood, gene fusion, and cooccurrence. The result was downloaded as a TSV format and imported into Cytoscape for visualization. The MCODE plug-in was used to identify the submodes from the PPI network. The Cytoscape plug-in MCODE (molecular complex detection) [29] was used to identify highly connected subclusters of proteins using a node score cutoff of 4 and node number cutoff of 5.

### 2.4. GO and KEGG Enrichment Analysis

KEGG pathway analysis and Gene Ontology (GO) analysis of CISD1 and its interacting proteins were performed using the clusterProfiler R package (v3.0.0) [30] with the FDR cutoff of 0.05. The results were visualized using the histogram generated by the “ggplot2” package [31].

### 2.5. CISD1 Promoter Methylation Analysis

Analysis of DNA methylation level of CISD1 promoter in HCC from TCGA was conducted using the UALCAN tool [31]. The DNA methylation data of patients with HCC was downloaded from the GEO datasets (GSE54503), and the DNA methylation level of the CISD1 promoter was verified by this dataset. DNA methylation is catalyzed by the DNA methyltransferase family. Therefore, we analyzed the correlation between CISD1 expression and the expression of four methyltransferases (DNMT1, DNMT2, DNMT3A, and DNMT3B) using TCGA LIHC gene expression data.

### 2.6. TIMER Database Analysis

The TIMER [32] database was used for analysis and visualization of the abundance of tumor-infiltrating immune cells (https://cistrome.shinyapps.io/timer/). The correlation analysis was evaluated in the TIMER database using Spearman's correlation analysis. We analyzed the correlation of CISD1 expression with the abundance of immune infiltrates, including B cells, CD4+ T cells, CD8+ T cells, neutrophils, macrophages, and dendritic cells, via gene modules.

## 3. Result

### 3.1. The mRNA Expression Level of CISD1 in Human Pan-Cancer

**Figure 1:**
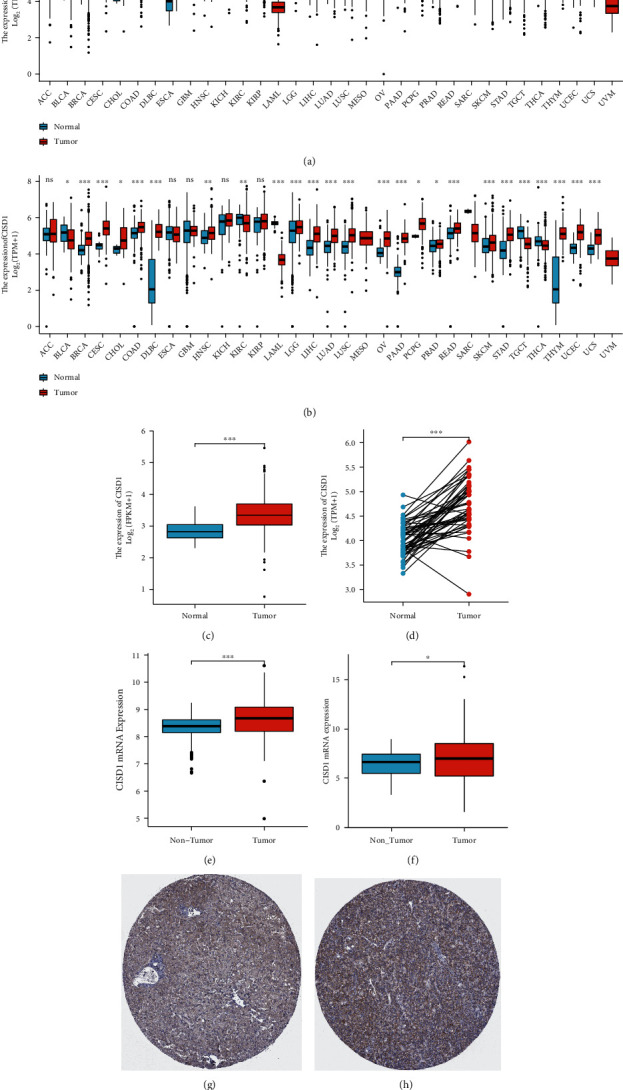
The mRNA expression level of CISD1 in human pan-cancer. (a) Increased or decreased CISD1 mRNA expression in human pan-cancer data of TCGA. (b) Increased or decreased CISD1 mRNA expression in human pan-cancer data of integrated TCGA and GETx. (c) The mRNA expression of CISD1 was significantly higher in LICH samples than in the unpaired nontumor samples. (d) The mRNA expression of CISD1 was significantly higher in LICH samples than in the paired nontumor samples. (e, f) Increased CISD1 mRNA expression in datasets of hepatocarcinomas compared with normal tissues in the Oncomine database. Immunohistochemical results of the HPA database showed that CISD1 protein was (g) moderately expressed in hepatocytes and (h) strongly expressed in hepatocarcinoma cells.

The mRNA expression of CISD1 in multiple cancers and tumor-adjacent normal tissues was analyzed using TCGA database data to detect the difference in expression of CISD1 in tumor and normal tissue. The result revealed that the CISD1 expression was higher in breast cancer, cholangiocarcinoma, colon cancer, esophageal cancer, head and neck squamous cell carcinoma, hepatocellular carcinoma, lung cancer, gastric cancer, and endometrial adenocarcinoma compared to the normal tissues. In addition, lower expression was observed in bladder cancer, brain cancer, kidney cancer, and thyroid cancer in the same datasets (Figure 1(a)). Considering the small number of normal samples in TCGA, we integrated the data of normal tissue in the GTEx database and the data of TCGA tumor tissues to analyze the expression differences of CISD1 in 27 different tumors (Figure 1(b)). The results of the previous analysis were confirmed. In addition, CISD1 expression was also significantly lower in myeloma, skin cancer, and testicular cancer compared with normal tissues and significantly higher in cervical squamous cell carcinoma, ovarian cancer, prostate cancer, and uterus cancer compared with normal tissues.

To further evaluate CISD1 expression in hepatocellular carcinoma, we detected the different expressions of CISD1 between hepatocellular carcinoma and normal tissue with TCGA LIHC data and two microarray expression data in GEO (GSE14520, GSE25097). The data showed a significantly high expression of CISD1 in HCC between unpaired and paired samples test (Figures 1(c) and 1(d)). Both GEO datasets showed that CISD1 expression was significantly higher in hepatocellular carcinoma than in normal tissue (Figures 1(e) and 1(f)).

To further determine the significance of CISD1 expression, the HPA (Human Protein Atlas) database was used to explore the difference of protein levels expression of CISD1 between HCC and normal liver tissues. The protein expression of CISD1 in HCC was higher than that in hepatocytes (Figures 1(g) and 1(h)).

### 3.2. Prognostic Value of CISD1 in Human Pan-Cancer

**Figure 2:**
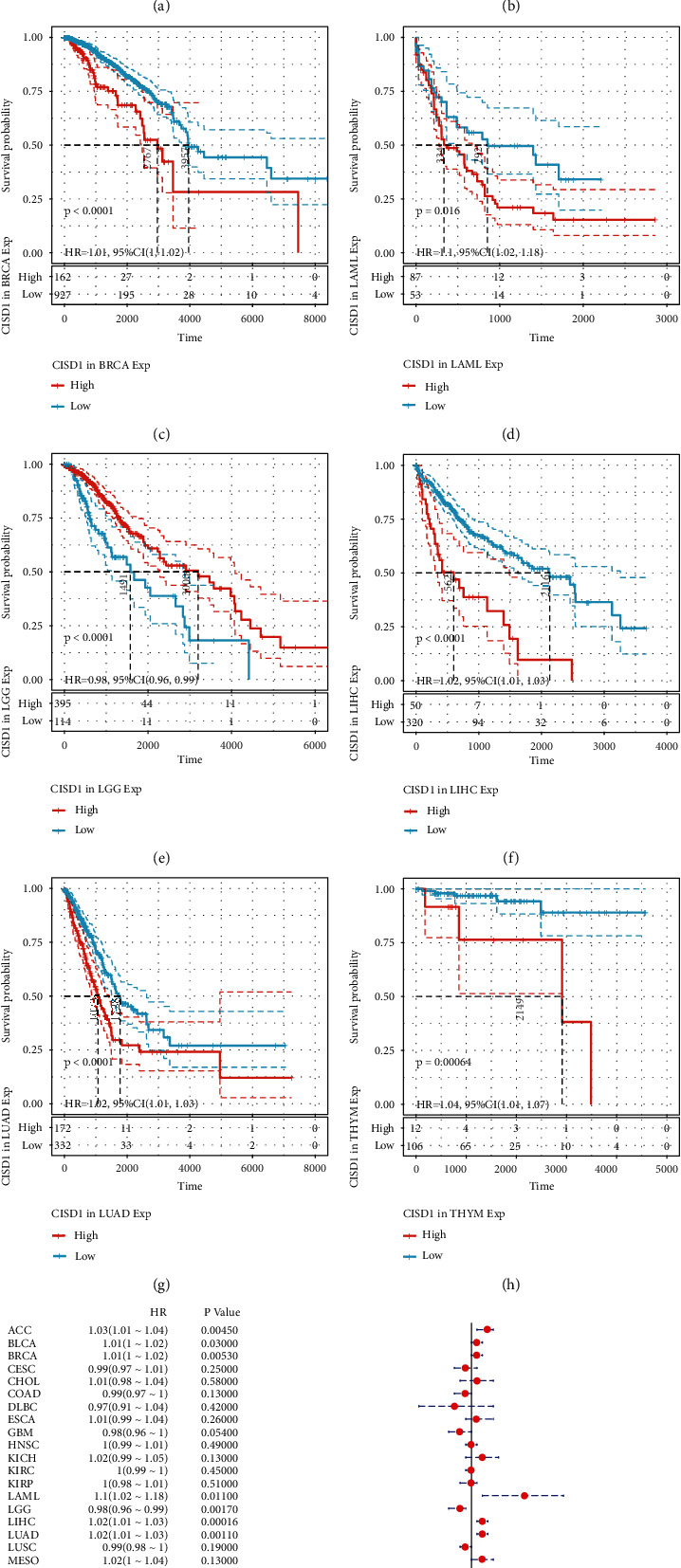
CISD1 expression and correlation with overall survival (OS) in different cancers. The Kaplan-Meier analysis of CISD1 expression in the (a) ACC, (b) BLCA, (c) BRCA, (d) LAML, (e) LGG, (f) LIHC, (g) LUAD, and (h) THYM. (i) The Forest plot illustrating the univariate Cox regression analysis of the prognostic impact of CISD1 expression on OS in 33 “pan-cancer” forms.

We investigated the prognostic value of CISD1 in human cancers. The impact of CISD1 expression on survival rates was analyzed using TCGA pan-cancer expression and clinical data by the univariate Cox regression analysis and Kaplan-Meier analysis. The results revealed that high CISD1 expression was significantly associated with poor OS of ACC (*HR*: 1.03 (1.01−1.04), *p* = 0.00450), BLCA (*HR*: 1.01 (1–1.02), *p* = 0.03), BRCA (*HR*: 1.01 (1–1.02), *p* = 0.0053), LAML (*HR*: 1.1 (1.02−1.18), *p* = 0.011), LIHC (*HR*: 1.02 (1.01−1.03), *p* = 0.00016), LUAD (*HR*: 1.02 (1.01−1.03), *p* = 0.0011), and THYM (*HR*: 1.04 (1.01−1.07), *p* = 0.0014), but with better OS in LGG (*HR*: 0.98 (0.96−0.99), *p* = 0.00170) (Figures 2(a)–2(i)). Similarly, overexpression of CISD1 was correlated with the poor DFS in LIHC (*HR*: 1.02 (1.01−1.03), *p* = 0.0037), but with better DFS in LGG (*HR*: 0.93 (0.89−0.98), *p* = 0.0035) (Supplementary Figure 1 A-C). Poorer DSS in ACC (*HR*: 1.03 (1.01−1.05), *p* = 0.0039), BLCA (*HR*: 1.01 (1 −1.03), *p* = 0.0036), LIHC (*HR*: 1.02 (1.01−1.03), *p* = 0.0031), LUAD (*HR*: 1.02 (1.01−1.04), *p* = 0.007), and UCEC (*HR*: 1.02 (1 −1.05), *p* = 0.002) and better DSS in LGG (*HR*: 0.97 (0.95−0.98), *p* = 0.0002) were shown to correlate with higher CISD1 expression (Supplementary Figure 2 A-G). The results also showed that overexpression of CISD1 was associated with poor PFS in ACC (*HR*: 1.03 (1.01−1.04), *p* = 0.00036), LIHC (*HR*: 1.02 (1.01−1.03), *p* = 0.0016), LUAD (*HR*: 1.01 (1–1.03), *p* = 0.021), SKCM (*HR*: 1.01 (1–1.02), *p* = 0.011), and UCEC (*HR*: 1.02 (1–1.03), *p* = 0.0031), but with better PFS in LGG (*HR*: 0.98 (0.96−0.99), *p* = 0.00015) (Supplementary Figure 3 A-G). These findings indicate that high CISD1 mRNA expression is associated with a poorer prognosis of OS, DFS, DSS, and PFS in LIHC patients but with better OS, DFS, DSS, and PFS in LGG patients.

**Figure 3:**
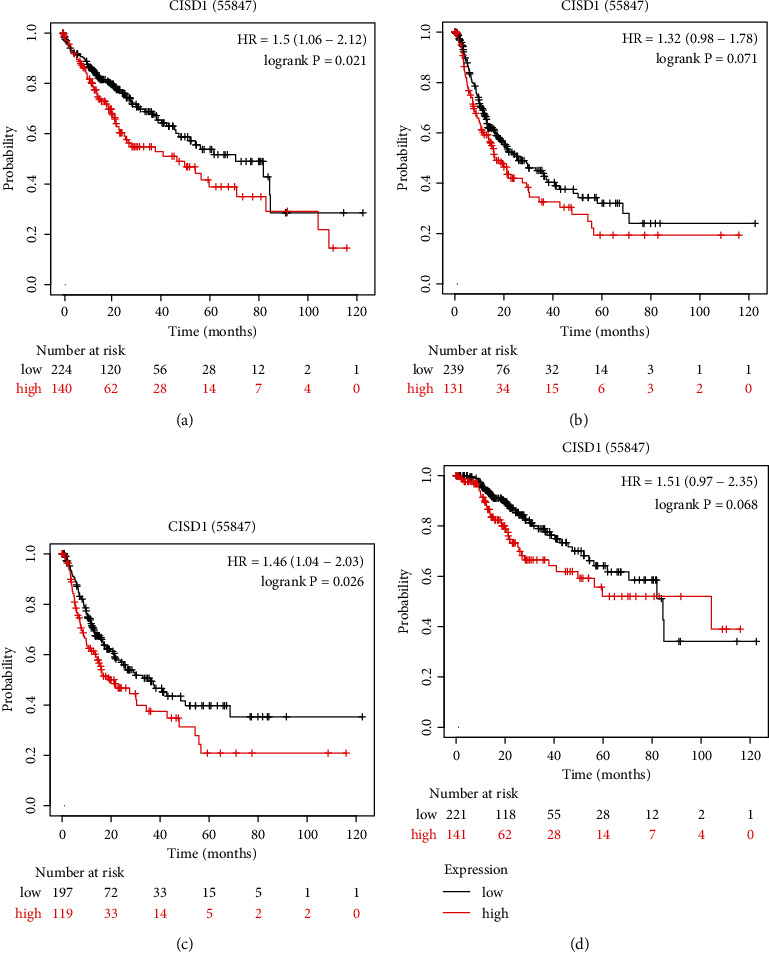
The Kaplan-Meier survival curves comparing the high and low expressions of CISD1 in liver cancer Kaplan-Meier plotter databases. (a) OS survival curves, (b) PFS survival curves, (c) RFS survival curves, and (d) DSS survival curves in liver cancer.

We further examined the prognostic value of CISD1 in hepatocellular carcinoma patients using the Kaplan-Meier plotter database (http://kmplot.com/analysis/) with Affymetrix microarrays data [33]. Disease-specific survival, disease-free survival, relapse-free survival, and overall survival were analyzed using the Kaplan-Meier survival analysis. The results showed that higher CISD1 mRNA expression was correlated with the poor prognosis in liver cancer (OS HR = 1.5 (1.06−2.12), *p* = 0.021; PFS HR = 1.32 (0.98−1.78), *p* = 0.071; RFS HR = 1.46 (1.04−2.03), *p* = 0.026; and DSS HR = 1.51 (0.97−2.35), *p* = 0.068) (Figures 3(a)–3(d)).

### 3.3. High mRNA Expression of CISD1 Impacts the Prognosis of Hepatocellular Carcinoma Patients Treated with Sorafenib

To explore the relationship between CISD1 mRNA expression level and clinicopathological characteristics of patients with HCC, we analyzed the impact of CISD1 expression level on prognosis in subgroups with different clinicopathological characteristics in the Kaplan-Meier plotter databases. Overexpression of the CISD1 was correlated with poor survival in male HCC patients (OS HR = 1.85 (1.19-2.88), *p* = 0.0059; PFS HR = 1.49 (1.03-2.15), *p* = 0.032) and T3 stage HCC patients (OS HR = 2.51 (0.98-6.43), *p* = 0.047; PFS HR = 2.68 (1.2-6.43), *p* = 0.013) as shown in Table 1. In addition, high expression of CISD1 was associated with worse OS in Asian HCC patients (OS HR = 1.87 (1.03-3.38), *p* = 0.035), stage I and II HCC patients (OS HR = 1.64 (1.02-2.67), *p* = 0.047), and patients with hepatitis virus-positive (OS HR = 2.12 (1.11-4.05), *p* = 0.019) (Table 1). However, high expression of CISD1 impacts the OS of none alcoholic patients (OS HR = 1.7 (1.05-2.07), *p* = 0.028) compared with alcoholic HCC patients (OS HR = 1.64 (0.85-3.12), *p* = 0.13) (Table 1). In the sorafenib treatment subgroups, lower expression of CISD1 was correlated with better OS (HR = 5.37 (1.37-21.12), *p* = 0.0081) and PFS (HR = 6.42 (2.37-17.4), *p* = 5.60*E* − 05) (Table 1).

### 3.4. PPI and Function Enrichment Analysis of CISD1

**Figure 4:**
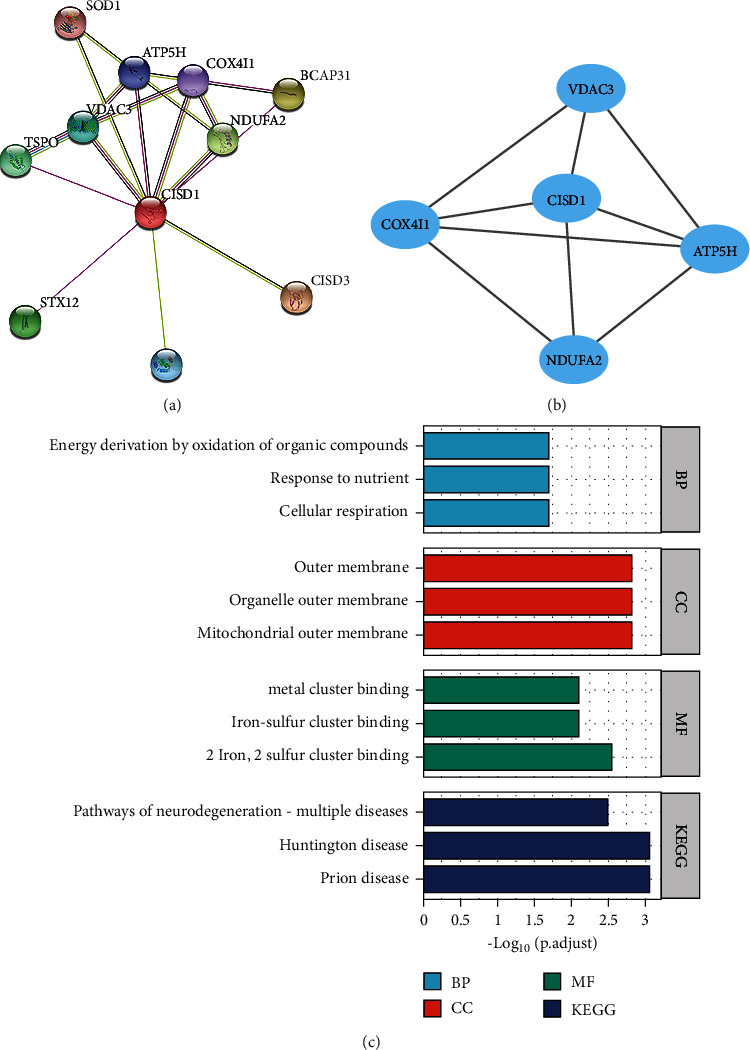
PPI and function enrichment analysis of CISD1. (a) PPI network of CISD1 (PPI enrichment *p* value = 0.0175). (b) MCODE modules (score = 4.5) of the PPI network. (c) Significant enriched GO term and KEGG pathways of CISD1 and its interacting proteins.

Single-protein PPI network analysis was performed by the STRING tool. There were 18 edges and 11 nodes in the PPI network (PPI enrichment *p* value = 0.0175) (Figure 4(a)). One MCODE module (score = 4.5) including 5 nodes was identified from the PPI network (Figure 4(b)). The GO and KEGG enrichment analyses of CISD1 and its interacting genes were performed by the clusterProfiler package. The results are shown in Figure 4(c) and Supplementary Table S1. The significant KEGG pathways were the Prion disease, Huntington disease, pathways of neurodegeneration in multiple diseases, cholesterol metabolism, and Parkinson's disease. The significant GO terms enriched in BP were cellular respiration, response to nutrients, energy derivation by oxidation of organic compounds, ATP metabolic process, and purine ribonucleoside triphosphate metabolic process. The significant GO terms enriched in CC were mitochondrial outer membrane, organelle outer membrane, outer membrane, an integral component of synaptic vesicle membrane, and mitochondrial membrane part. The significant GO terms enriched in MF were 2 iron-2 sulfur cluster binding, iron-sulfur cluster binding, metal cluster binding, benzodiazepine receptor activity, and voltage-gated anion channel activity.

### 3.5. Gene Epigenetic Regulation Leads to High Expression of CISD1 in HCC

**Figure 5:**
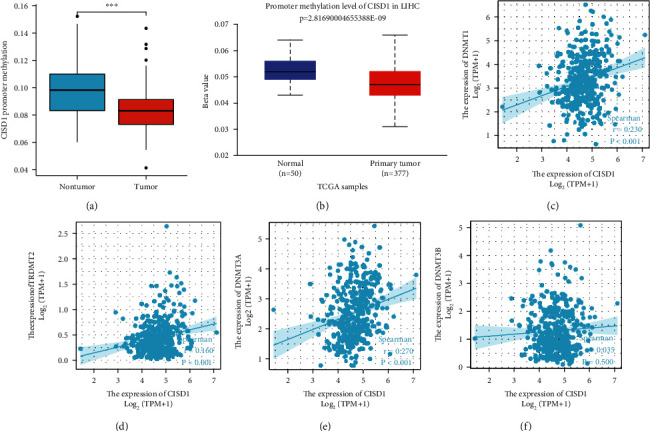
Gene epigenetic regulation leads to high expression of CISD1 in HCC. (a) Decreased CISD1 promoter methylation in datasets of hepatocarcinomas compared with normal tissues in the Oncomine database. (b) Methylation of the CISD1 promoter is lower in HCC than that in normal tissue in TCGA LICH dataset collected by UALCAN. (c) mRNA expression of CISD1 is positively correlated with DNMT1 mRNA expression in TCGA LIHC cohort. (d) mRNA expression of CISD1 is positively correlated with DNMT2 mRNA expression in TCGA LIHC cohort. (e) mRNA expression of CISD1 is positively correlated with DNMT3A mRNA expression in TCGA LIHC cohort. (f) mRNA expression of CISD1 is not related to DNMT3B mRNA expression in TCGA LIHC cohort.

In general, hypermethylation of the gene's promoter regions results in low expression of the gene, while hypomethylation of the gene's promoter regions results in high expression of the gene [34]. To explore the methylation status of the promoter region of the CISD1 gene, an analysis of the DNA methylation level of CISD1 promoter in HCC from TCGA was conducted by the UALCAN tool. The results showed that methylation of the promoter of CISD1 is lower in HCC than that in normal tissue and the mRNA expression of CISD1 was high in HCC (Figure 5(b)). We also verified the differentially methylated CpG sites (DMCs) between HCC and liver tissue in GEO datasets GSE54503. As shown in Figure 5(a), methylation of the promoter of CISD1 is significantly lower in HCC than that in liver tissue. DNA methylation is catalyzed by the DNA methyltransferase family. Therefore, we analyzed the correlation between CISD1 expression and the expression of four methyltransferases (DNMT1, DNMT2, DNMT3A, and DNMT3B). mRNA expression of CISD1 is significantly positively correlated with DNMT1, DNMT2, and DNMT3A mRNA expressions (Figures 5(c)–5(f)).

### 3.6. CISD1 Expression Is Correlated with Immune Cell Infiltration Level in Hepatocellular Carcinoma

**Figure 6:**
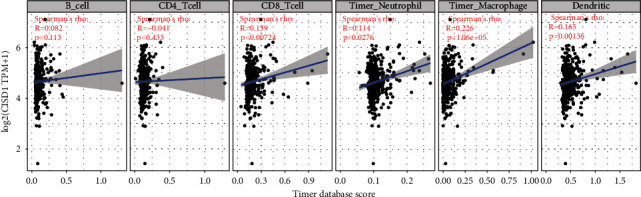
CISD1 expression has significant correlations with infiltrating levels of CD8 T cells, neutrophils, macrophages, and dendritic cells in HCC.

We analyzed the correlation of CISD1 expression with the abundance of immune infiltrates, including B cells, CD4+ T cells, CD8+ T cells, neutrophils, macrophages, and dendritic cells, via gene modules in the TIMER database. The results showed that CISD1 expression was significantly correlated with infiltrating levels of CD8+ T cells (*r* = 0.139, *p* = 0.00724), neutrophils (*r* = 0.114, *p* = 0.0276), macrophages (*r* = 0.226, *p* = 1.06*E* − 05), and dendritic cells (*r* = 0.165, *p* = 0.00136) in liver cancer (Figure 6).

## 4. Discussion

HCC is a leading cause of cancer-related death in many regions of the world [35]. In the last few decades, considerable research progress has been made in the epidemiology, risk factors, and molecular characteristics of HCC. However, the specific molecular mechanism of HCC remains unclear and needs to be further explored. Recent studies revealed that the CISD1 plays a critical role in promoting the proliferation of cancer cells, supporting tumor growth and metastasis [36]. But the role of CISD1 in HCC remains unclear. In this study, we mined public databases and used bioinformatics analysis to reveal that CISD1 mRNA is overexpressed in HCC than that in the liver cell and that high expression of the CISD1 is correlated with poor prognosis. Interestingly, high mRNA expression of CISD1 can impact the prognosis of hepatocellular carcinoma patients who were treated with sorafenib indicating that the CISD1 antagonist may enhance the anti-HCC effect of sorafenib. Furthermore, our analysis showed that in HCC, CD8+ T cell, neutrophil, macrophage, and dendritic cell infiltration levels were correlated with levels of the CISD1 expression. Thus, our study provides insights into understanding the potential role of CISD1 in tumor immunology and its use as potential anticancer targets.

Differential expression is a prerequisite for genes to play a role in tumorigenesis and tumor development. Hence, this study first examined the differential expression of CISD1 in various tumors and corresponding normal tissues using independent datasets in TCGA and GEO. The differential expression of CISD1 between cancer and normal tissues was observed in many types of cancers. Because the sample size of normal tissue from TCGA database was small, we integrated the RNA-seq data of GTEx [36] and TCGA to improve the reliability of the results. This study revealed that the CISD1 expression was higher in breast cancer, cholangiocarcinoma, colon cancer, esophageal cancer, head and neck squamous cell carcinoma, hepatocellular carcinoma, lung cancer, gastric cancer, and endometrial adenocarcinoma compared to the normal tissues. Lower expression was observed in bladder cancer, brain cancer, kidney cancer, and thyroid cancer. To further investigate the expression of CISD1 in liver cancer, we verified the expression of CISD1 at mRNA and protein levels using the GEO dataset (GSE14520, GSE25097) and HPA database, respectively. Hypomethylation in the promoter may cause increased gene expression. The results of methylation status showed that methylation of the promoter of CISD1 was lower in HCC than that in normal tissue and the mRNA expression of CISD1 was high in HCC. We also verified the differentially methylated CpG sites (DMCs) between HCC and liver tissue in GEO datasets (GSE54503). These results indicated that epigenetic regulation may cause high expression of CISD1 in HCC. The impact of CISD1 expression on survival rates was analyzed, and results showed that high CISD1 mRNA expression was associated with a poorer prognosis of OS, DFS, DSS, and PFS in LIHC patients but with better OS, DFS, DSS, and PFS in LGG patients. In addition, overexpression of the CISD1 was correlated with poor survival in males, T3 stage (TNM classification), stage I and II HCC patients, and hepatitis virus-positive patients. High expression of CISD1 negatively impacts the OS of nonalcoholic patients compared with alcoholic HCC patients. These changes may be related due to some ferroptosis-related genes expressions as previous studies in different cancer types have shown 19 ferroptosis-related genes as a potential biomarker of OS in glioma patients [37] and 10-ferroptosis-related gene signature for a prognosis for patients with hepatocellular carcinoma [38].

These findings strongly suggest that CISD1 is a prognostic biomarker in hepatocellular carcinoma. Interestingly, stratified analysis by clinical characteristics of liver cancer patients showed that patients with low CISD1 expression had a better prognosis after treatment with sorafenib. It is probably because that CISD1 can inhibit ferroptosis by protecting against mitochondrial lipid peroxidation. Recently, Li et al. have reported that MTX1 upregulation in HCC contributed to sorafenib resistance possibly involving CISD1-mediated autophagy mechanisms [17].

To explore the function of CISD1 in cells, PPI and the GO/KEGG pathway analysis were performed with the help of bioinformatics. One MCODE module (score = 4.5) including 5 nodes was identified from the PPI network. These gene nodes include CISD1, ATP5H, NDUFA2, COX4I1, and VDAC3, and all of them are associated with the mitochondrial respiratory chain. The GO analysis also showed significant terms enriched in BP which were cellular respiration and energy metabolism, significant terms enriched in CC were mitochondrial outer membrane, and significant GO terms enriched in MF were 2 iron-2 sulfur cluster binding and iron-sulfur cluster binding. Over the past decade, researchers have identified “iron dependence” as a key phenotype in cancer cells with unknown mechanisms [39, 40]. It has been suggested that cancer cells need excess iron to support their high metabolic rates because iron is a cofactor in many different proteins that participate in DNA and protein synthesis, glycolysis, and cell respiration [41]. Cancer cells exhibit iron- and ROS-dependent phenotypes. Combined with the regulation of CISD1 protein on iron and ROS metabolism in mitochondria and the high expression level of CISD1 protein in different tumors, CISD1 protein may play a key role in cancer cell proliferation [42].

In recent years, immunotherapy has gradually emerged as the most promising method for the treatment of cancer. Immune cells infiltrated in the tumor microenvironment have a quite crucial influence on the occurrence and development of tumors [43]. We analyzed the correlation of CISD1 expression with the abundance of immune infiltrates, including B cells, CD4+ T cells, CD8+ T cells, neutrophils, macrophages, and dendritic cells, via gene modules in the TIMER database. The results showed that CISD1 expression has positive correlations with infiltrating levels of CD8+ T cells, neutrophils, macrophages, and dendritic cells in liver cancer. The cellular components of the tumor microenvironment (TME) are rather complex and very different from the microenvironment of normal tissue. The tumor microenvironment influences neoplastic progression and growth [44]. The myeloid cells and lymphocytes, as the major cellular components in the tumor microenvironment, play important roles in inflammation, cancer immune evasion, and responses to immunotherapy treatment [45]. The majority of studies suggest that liver macrophages can promote inflammation and tumor cell development and inhibit antitumor immunity [46]. Dendritic cells (DC) are professional antigen-presenting cells, while T cells are efficient antitumor effector cells. Insufficient cross talk between DCs and T cells is one of the main mechanisms of HCC tumor tolerance [47]. Therefore, CISD1 expression may impact the prognosis via increasing immune infiltration levels in CD8+ T cells, macrophages, neutrophils, and DCs in HCC.

In summary, CISD1 is overexpressed in multiple human tumors including HCC. CISD1 expression was significantly higher in hepatocellular carcinoma than in normal tissue and hepatocytes. Overexpression of the CISD1 in HCC may be caused by hypomethylation in the gene promoter. Increased CISD1 expression is associated with poor prognosis and increased immune infiltration levels in CD8+ T cells, macrophages, neutrophils, and DCs of hepatocarcinoma. Therefore, CISD1 may serve as a potential prognostic biomarker in patients with HCC.

## Figures and Tables

**Table 1:** Correlation of CISD1 mRNA expression and clinical prognosis in liver cancer with different clinicopathological factors by the Kaplan-Meier plotter.

Clinicopathological characteristics	Overall survival (*n* = 364)			Progression-free survival (*n* = 370)		
	*N*	Hazard ratio	*p* value	*N*	Hazard ratio	*p* value
Sex						
Female	121	0.66 (0.37-1.17)	0.15	121	0.86 (0.49-1.49)	0.58
Male	250	1.85 (1.19-2.88)	*0.0059*	250	1.49 (1.03-2.15)	*0.032*
Stage						
I+II	257	1.64 (1.02-2.67)	*0.047*	257	1.24 (0.84-1.83)	0.28
III+IV	90	1.85 (0.86-3.99)	0.11	90	2.02 (1.01-4.01)	*0.041*
Stage T						
1	180	1.57 (0.87-2.85)	0.13	180	0.73 (0.44-1.23)	0.24
2	94	0.55 (0.25-1.88)	0.12	94	0.6 (0.32-1.18)	0.11
3	78	2.51 (0.98-6.43)	*0.047*	78	2.68 (1.2-6.43)	*0.013*
Grade						
1	55	0.56 (0.2-1.58)	0.27	55	1.81 (0.72-4.54)	0.2
2	177	0.16 (0.86-2.42)	0.16	177	1.57 (1.01-2.44)	*0.043*
3	122	1.64 (0.9-2.98)	0.11	122	1.33 (0.75-2.34)	0.32
Race						
White	184	0.75 (0.47-1.2)	0.23	184	1.33 (0.89-1.98)	0.16
Asian	158	1.87 (1.03-3.38)	*0.035*	158	1.55 (0.86-2.8)	0.14
Sorafenib treatment						
Treated	30	5.37 (1.37-21.12)	*0.0081*		6.42 (2.37-17.4)	5.60*E* − 05
Hepatitis virus						
Yes	153	2.12 (1.11-4.05)	*0.019*	153	1.19 (0.71-2)	0.5
None	169	0.82 (0.52-1.3)	0.4	169	1.5 (0.97-2.31)	0.067
Alcohol consumption						
Yes	117	1.64 (0.85-3.12)	0.13	117	1.52 (0.91-2.56)	0.11
None	205	1.7 (1.05-2.07)	*0.028*	205	1.34 (0.87-2.08)	0.18

## Data Availability

The datasets analyzed for this study can be found in the UCSC Xena platform (https://xenabrowser.net/datapages/) and Gene Expression Omnibus (GEO) (https://www.ncbi.nlm.nih.gov/geo/).

## Supplementary Materials

Supplementary 1 Significant enriched GO term and KEGG pathways of CISD1 and its interacting proteins.

Supplementary 2 Gene expression of the CSID1 is significantly associated with disease-free survival in cancers.

Supplementary 3 Gene expression of the CSID1 is significantly associated with progression-free survival in cancers.

Supplementary 4 Gene expression of the CSID1 is significantly associated with disease-specific survival in cancers.
