# Supplementary material for: Overexpression of CISD1 Predicts Worse Survival in Hepatocarcinoma Patients
Source: Biomed Res Int. 2022 Mar 11;2022:7823191. doi: 10.1155/2022/7823191 (PMC8933656; doi:10.1155/2022/7823191)
Supplement: Supplementary 1 — Significant enriched GO term and KEGG pathways of CISD1 and its interacting proteins. [file 7823191.f1.docx]

| ONTOLOGY | ID | Description | GeneRatio | BgRatio | pvalue | p.adjust | qvalue |
| --- | --- | --- | --- | --- | --- | --- | --- |
| BP | GO:0045333 | cellular respiration | 3/9 | 193/18670 | 8.73e-05 | 0.020 | 0.008 |
| BP | GO:0007584 | response to nutrient | 3/9 | 219/18670 | 1.27e-04 | 0.020 | 0.008 |
| BP | GO:0015980 | energy derivation by oxidation of organic compounds | 3/9 | 285/18670 | 2.76e-04 | 0.020 | 0.008 |
| BP | GO:0046034 | ATP metabolic process | 3/9 | 305/18670 | 3.37e-04 | 0.020 | 0.008 |
| BP | GO:0009205 | purine ribonucleoside triphosphate metabolic process | 3/9 | 335/18670 | 4.44e-04 | 0.020 | 0.008 |
| CC | GO:0005741 | mitochondrial outer membrane | 3/9 | 178/19717 | 5.84e-05 | 0.002 | 5.19e-04 |
| CC | GO:0031968 | organelle outer membrane | 3/9 | 201/19717 | 8.38e-05 | 0.002 | 5.19e-04 |
| CC | GO:0019867 | outer membrane | 3/9 | 203/19717 | 8.63e-05 | 0.002 | 5.19e-04 |
| CC | GO:0030285 | integral component of synaptic vesicle membrane | 2/9 | 34/19717 | 1.03e-04 | 0.002 | 5.19e-04 |
| CC | GO:0044455 | mitochondrial membrane part | 3/9 | 229/19717 | 1.23e-04 | 0.002 | 5.19e-04 |
| MF | GO:0051537 | 2 iron, 2 sulfur cluster binding | 2/9 | 22/17697 | 5.28e-05 | 0.003 | 0.002 |
| MF | GO:0051536 | iron-sulfur cluster binding | 2/9 | 63/17697 | 4.42e-04 | 0.008 | 0.004 |
| MF | GO:0051540 | metal cluster binding | 2/9 | 63/17697 | 4.42e-04 | 0.008 | 0.004 |
| MF | GO:0008503 | benzodiazepine receptor activity | 1/9 | 11/17697 | 0.006 | 0.070 | 0.037 |
| MF | GO:0008308 | voltage-gated anion channel activity | 1/9 | 17/17697 | 0.009 | 0.070 | 0.037 |
| KEGG | hsa05020 | Prion disease | 4/7 | 273/8076 | 4.13e-05 | 8.72e-04 | 5.10e-04 |
| KEGG | hsa05016 | Huntington disease | 4/7 | 306/8076 | 6.46e-05 | 8.72e-04 | 5.10e-04 |
| KEGG | hsa05022 | Pathways of neurodegeneration - multiple diseases | 4/7 | 475/8076 | 3.59e-04 | 0.003 | 0.002 |
| KEGG | hsa04979 | Cholesterol metabolism | 2/7 | 50/8076 | 7.73e-04 | 0.005 | 0.003 |
| KEGG | hsa05012 | Parkinson disease | 3/7 | 249/8076 | 9.25e-04 | 0.005 | 0.003 |

**Table S1.** Significant enriched GO term and KEGG pathways of CISD1 and it interacting proteins.
