# Supplementary material for: Overexpression of CISD1 Predicts Worse Survival in Hepatocarcinoma Patients
Source: Biomed Res Int. 2022 Mar 11;2022:7823191. doi: 10.1155/2022/7823191 (PMC8933656; doi:10.1155/2022/7823191)

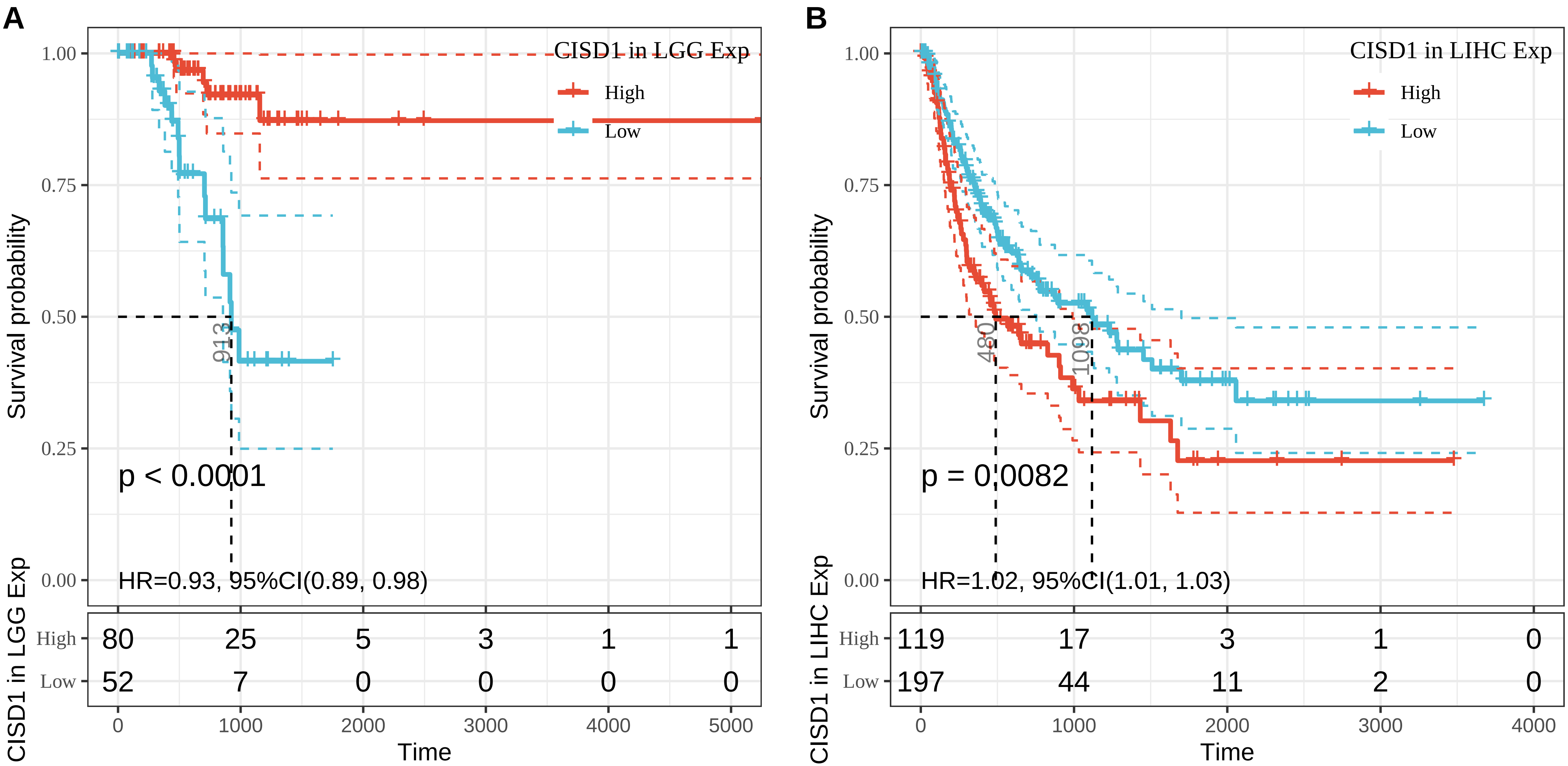
A
CISD1 in LGG Exp
High
Low
Survival probability
1.00
0.75
0.50
0.25
0.00
913
p < 0.0001
HR=0.93, 95%CI(0.89, 0.98)
CISD1 in LGG Exp
High 80 25 5 3 1 1
Low 52 7 0 0 0 0
0 1000 2000 3000 4000 5000
Time
B
CISD1 in LIHC Exp
High
Low
Survival probability
1.00
0.75
0.50
0.25
0.00
480
1098
p = 0.0082
HR=1.02, 95%CI(1.01, 1.03)
CISD1 in LIHC Exp
High 119 17 3 1 0
Low 197 44 11 2 0
0 1000 2000 3000 4000
Time

C

	HR	P Value
ACC	1.02(0.99 ~ 1.05)	0.1500
BLCA	1.01(0.98 ~ 1.04)	0.5500
BRCA	1(0.99 ~ 1.02)	0.6700
CESC	0.99(0.96 ~ 1.02)	0.5500
CHOL	1.02(0.98 ~ 1.06)	0.3400
COAD	1(0.97 ~ 1.03)	0.9400
DLBC	0.91(0.78 ~ 1.07)	0.2800
ESCA	1.01(0.97 ~ 1.06)	0.5000
GBM	NA(NA ~ NA)	
HNSC	1.02(0.99 ~ 1.05)	0.3300
KICH	1.03(0.97 ~ 1.09)	0.3200
KIRC	1.01(0.99 ~ 1.02)	0.4100
KIRP	1.01(1 ~ 1.02)	0.2000
LAML	NA(NA ~ NA)	
LGG	0.93(0.89 ~ 0.98)	0.0035
LIHC	1.02(1.01 ~ 1.03)	0.0037
LUAD	1.01(0.99 ~ 1.03)	0.5400
LUSC	1(0.98 ~ 1.02)	0.8600
MESO	1.02(0.9 ~ 1.15)	0.7800
OV	1.01(0.99 ~ 1.02)	0.3900
PAAD	0.98(0.93 ~ 1.02)	0.3100
PCPG	0.99(0.93 ~ 1.06)	0.8300
PRAD	0.95(0.89 ~ 1.02)	0.1700
READ	0.92(0.84 ~ 1.02)	0.1000
SARC	1(0.99 ~ 1.01)	0.9500
SKCM	NA(NA ~ NA)	
STAD	0.98(0.95 ~ 1.02)	0.3100
TGCT	1.01(0.96 ~ 1.07)	0.6100
THCA	0.95(0.88 ~ 1.01)	0.1200
THYM	NA(NA ~ NA)	
UCEC	1.01(0.99 ~ 1.03)	0.4900
UCS	0.97(0.92 ~ 1.03)	0.3600
UVM	NA(NA ~ NA)	

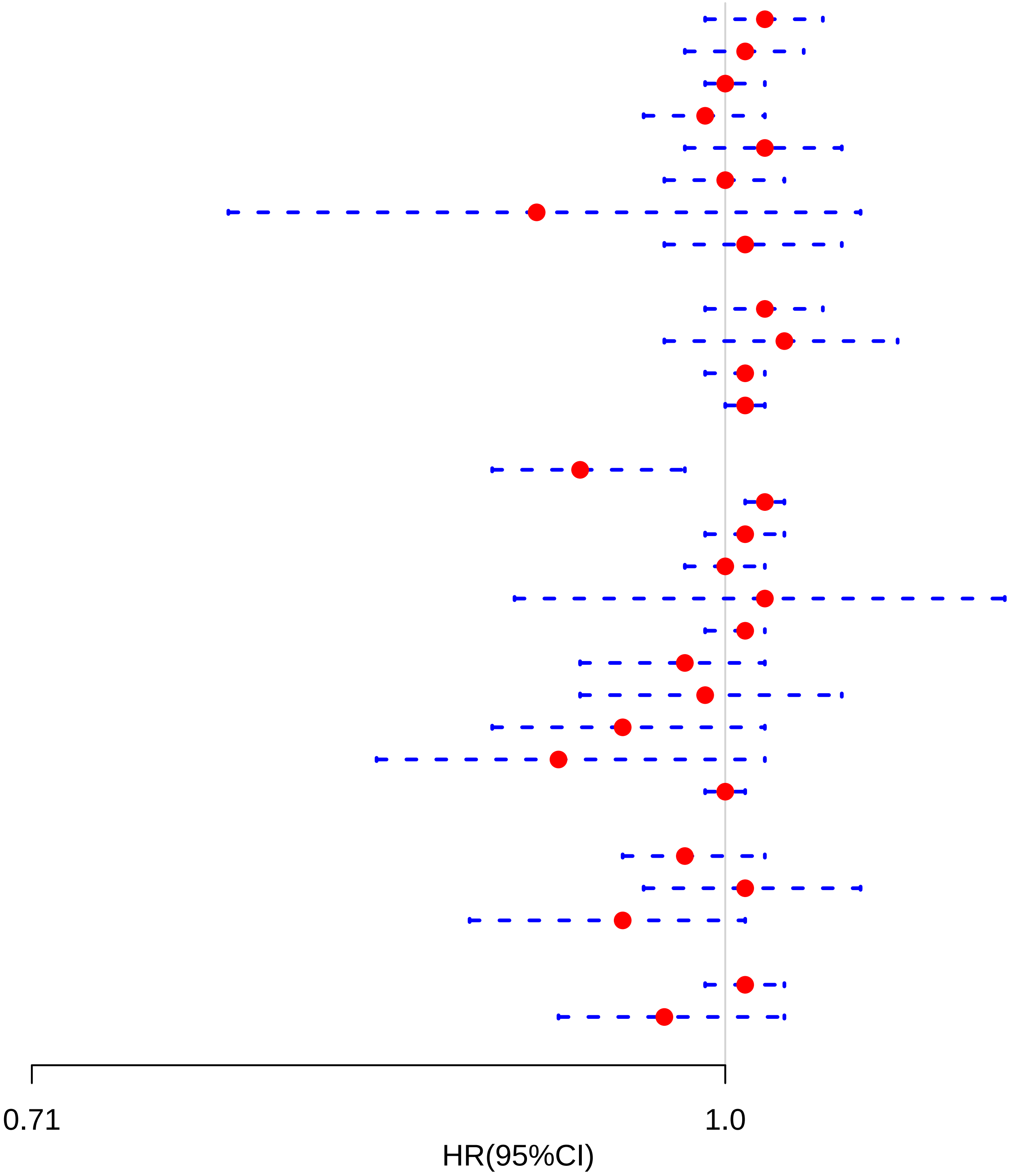

Supplement: Supplementary 2 — Gene expression of the CSID1 is significantly associated with disease-free survival in cancers. [file 7823191.f2.pdf]
