# Supplementary material for: Overexpression of CISD1 Predicts Worse Survival in Hepatocarcinoma Patients
Source: Biomed Res Int. 2022 Mar 11;2022:7823191. doi: 10.1155/2022/7823191 (PMC8933656; doi:10.1155/2022/7823191)
Supplement: Supplementary 3 — Gene expression of the CSID1 is significantly associated with progression-free survival in cancers. [file 7823191.f3.pdf]

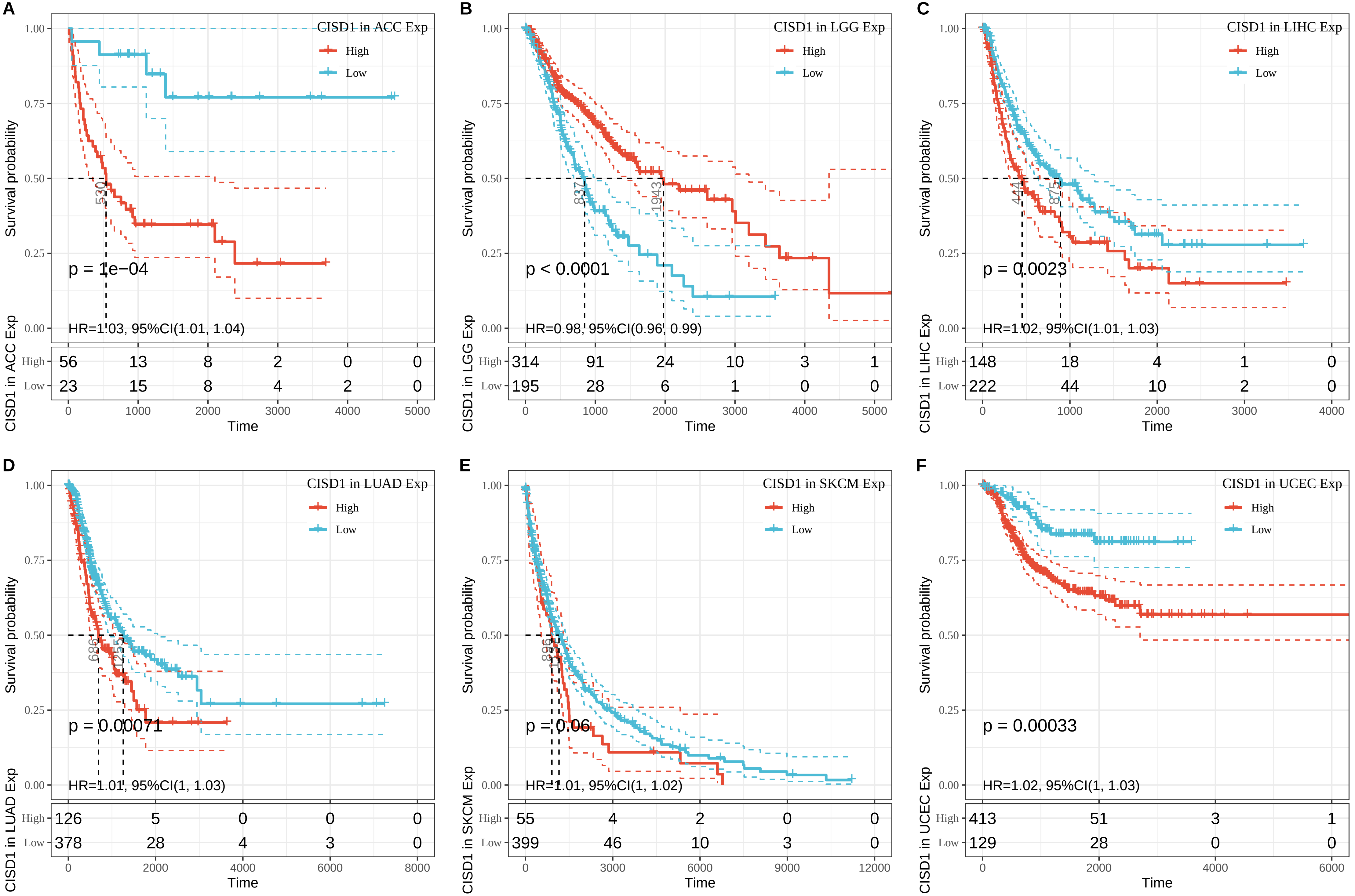
A
CISD1 in ACC Exp
High
Low
Survival probability
p = 1e−04
HR=1.03, 95%CI(1.01, 1.04)
530
CISD1 in ACC Exp
High 56 13 8 2 0 0
Low 23 15 8 4 2 0
Time
B
CISD1 in LGG Exp
High
Low
Survival probability
p < 0.0001
HR=0.98, 95%CI(0.96, 0.99)
837
1943
CISD1 in LGG Exp
High 314 91 24 10 3 1
Low 195 28 6 1 0 0
Time
C
CISD1 in LIHC Exp
High
Low
Survival probability
p = 0.0023
HR=1.02, 95%CI(1.01, 1.03)
444
875
CISD1 in LIHC Exp
High 148 18 4 1 0
Low 222 44 10 2 0
Time
D
CISD1 in LUAD Exp
High
Low
Survival probability
p = 0.00071
HR=1.01, 95%CI(1, 1.03)
686
1255
CISD1 in LUAD Exp
High 126 5 0 0 0
Low 378 28 4 3 0
Time
E
CISD1 in SKCM Exp
High
Low
Survival probability
p = 0.06
HR=1.01, 95%CI(1, 1.02)
896
CISD1 in SKCM Exp
High 55 4 2 0 0
Low 399 46 10 3 0
Time
F
CISD1 in UCEC Exp
High
Low
Survival probability
p = 0.00033
HR=1.02, 95%CI(1, 1.03)
CISD1 in UCEC Exp
High 413 51 3 1
Low 129 28 0 0
Time

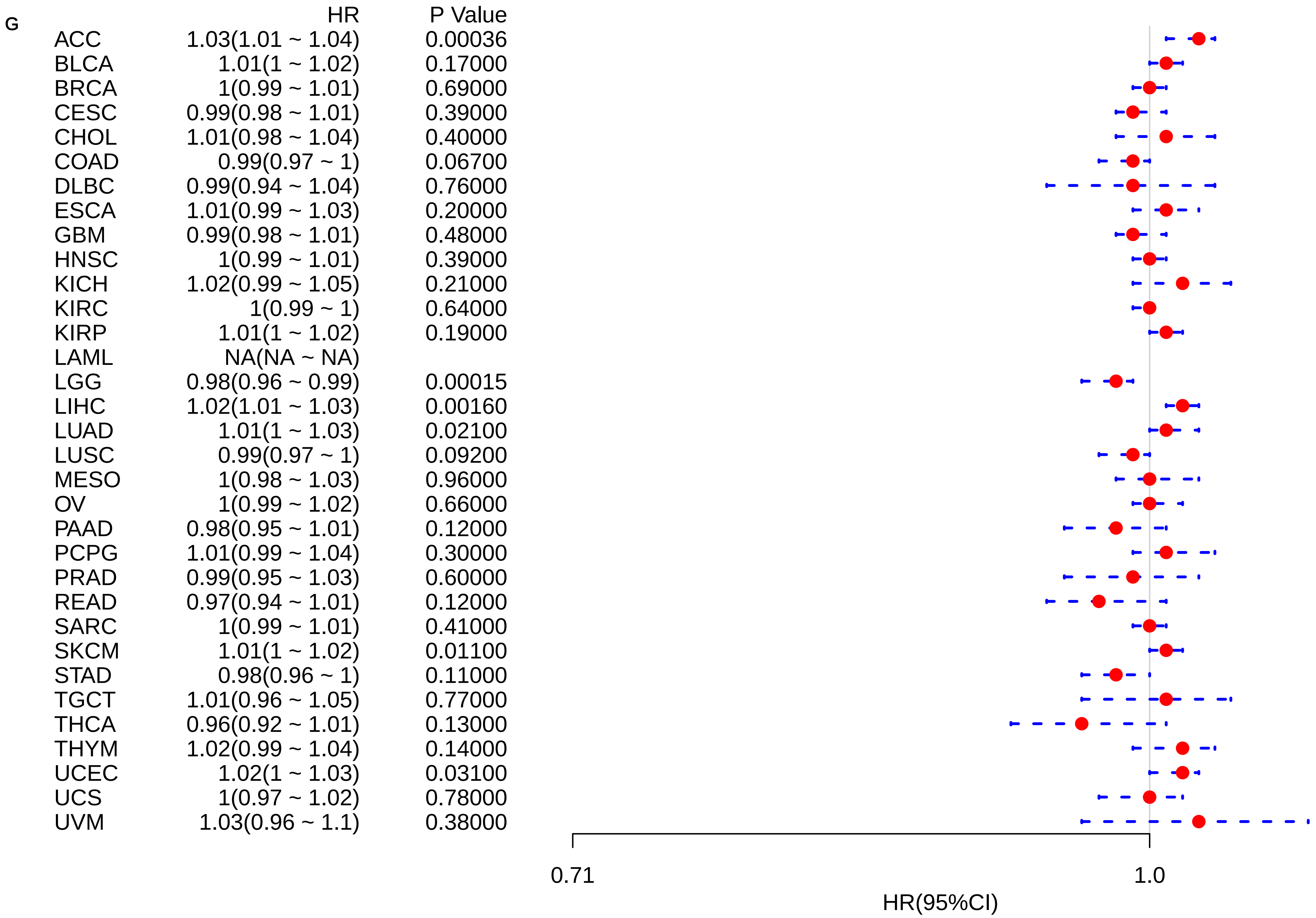
G
HR
P Value
ACC 1.03(1.01 ~ 1.04) 0.00036
BLCA 1.01(1 ~ 1.02) 0.17000
BRCA 1(0.99 ~ 1.01) 0.69000
CESC 0.99(0.98 ~ 1.01) 0.39000
CHOL 1.01(0.98 ~ 1.04) 0.40000
COAD 0.99(0.97 ~ 1) 0.06700
DLBC 0.99(0.94 ~ 1.04) 0.76000
ESCA 1.01(0.99 ~ 1.03) 0.20000
GBM 0.99(0.98 ~ 1.01) 0.48000
HNSC 1(0.99 ~ 1.01) 0.39000
KICH 1.02(0.99 ~ 1.05) 0.21000
KIRC 1(0.99 ~ 1) 0.64000
KIRP 1.01(1 ~ 1.02) 0.19000
LAML NA(NA ~ NA)
LGG 0.98(0.96 ~ 0.99) 0.00015
LIHC 1.02(1.01 ~ 1.03) 0.00160
LUAD 1.01(1 ~ 1.03) 0.02100
LUSC 0.99(0.97 ~ 1) 0.09200
MESO 1(0.98 ~ 1.03) 0.96000
OV 1(0.99 ~ 1.02) 0.66000
PAAD 0.98(0.95 ~ 1.01) 0.12000
PCPG 1.01(0.99 ~ 1.04) 0.30000
PRAD 0.99(0.95 ~ 1.03) 0.60000
READ 0.97(0.94 ~ 1.01) 0.12000
SARC 1(0.99 ~ 1.01) 0.41000
SKCM 1.01(1 ~ 1.02) 0.01100
STAD 0.98(0.96 ~ 1) 0.11000
TGCT 1.01(0.96 ~ 1.05) 0.77000
THCA 0.96(0.92 ~ 1.01) 0.13000
THYM 1.02(0.99 ~ 1.04) 0.14000
UCEC 1.02(1 ~ 1.03) 0.03100
UCS 1(0.97 ~ 1.02) 0.78000
UVM 1.03(0.96 ~ 1.1) 0.38000
0.71
1.0
HR(95%CI)
