# Supplementary material for: Overexpression of CISD1 Predicts Worse Survival in Hepatocarcinoma Patients
Source: Biomed Res Int. 2022 Mar 11;2022:7823191. doi: 10.1155/2022/7823191 (PMC8933656; doi:10.1155/2022/7823191)

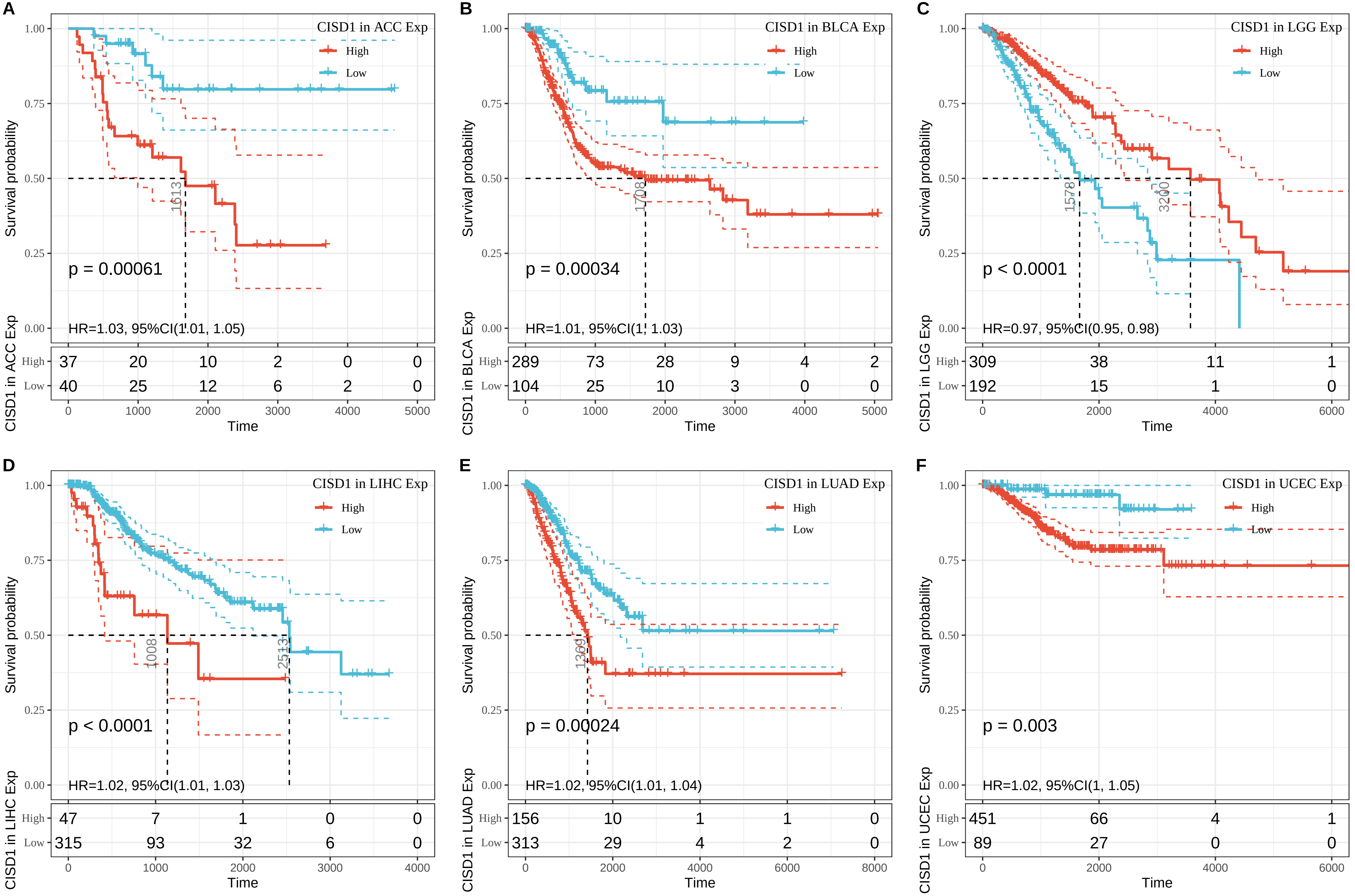
A
CISD1 in ACC Exp
High
Low
Survival probability
1.00
0.75
0.50
0.25
0.00
1613
p = 0.00061
HR=1.03, 95%CI(1.01, 1.05)
CISD1 in ACC Exp
High 37 20 10 2 0 0
Low 40 25 12 6 2 0
0 1000 2000 3000 4000 5000
Time
B
CISD1 in BLCA Exp
High
Low
Survival probability
1708
p = 0.00034
HR=1.01, 95%CI(1, 1.03)
CISD1 in BLCA Exp
High 289 73 28 9 4 2
Low 104 25 10 3 0 0
0 1000 2000 3000 4000 5000
Time
C
CISD1 in LGG Exp
High
Low
Survival probability
1578
3200
p < 0.0001
HR=0.97, 95%CI(0.95, 0.98)
CISD1 in LGG Exp
High 309 38 11 1
Low 192 15 1 0
0 2000 4000 6000
Time
D
CISD1 in LIHC Exp
High
Low
Survival probability
1008
2513
p < 0.0001
HR=1.02, 95%CI(1.01, 1.03)
CISD1 in LIHC Exp
High 47 7 1 0 0
Low 315 93 32 6 0
0 1000 2000 3000 4000
Time
E
CISD1 in LUAD Exp
High
Low
Survival probability
1369
p = 0.00024
HR=1.02, 95%CI(1.01, 1.04)
CISD1 in LUAD Exp
High 156 10 1 1 0
Low 313 29 4 2 0
0 2000 4000 6000 8000
Time
F
CISD1 in UCEC Exp
High
Low
Survival probability
p = 0.003
HR=1.02, 95%CI(1, 1.05)
CISD1 in UCEC Exp
High 451 66 4 1
Low 89 27 0 0
0 2000 4000 6000
Time

G

	HR	P Value
ACC	1.03(1.01 ~ 1.05)	0.0039
BLCA	1.01(1 ~ 1.03)	0.0360
BRCA	1.01(1 ~ 1.03)	0.0660
CESC	0.99(0.97 ~ 1.01)	0.3800
CHOL	1.01(0.98 ~ 1.04)	0.4200
COAD	0.98(0.97 ~ 1)	0.1200
DLBC	1(0.93 ~ 1.08)	0.9800
ESCA	1.02(0.99 ~ 1.05)	0.1800
GBM	0.98(0.96 ~ 1)	0.0800
HNSC	1(0.99 ~ 1.02)	0.7100
KICH	1.03(0.99 ~ 1.06)	0.1500
KIRC	1(0.99 ~ 1.01)	0.5000
KIRP	1(0.98 ~ 1.02)	0.8200
LAML	NA(NA ~ NA)	
LGG	0.97(0.95 ~ 0.98)	0.0002
LIHC	1.02(1.01 ~ 1.03)	0.0031
LUAD	1.02(1.01 ~ 1.04)	0.0070
LUSC	0.98(0.96 ~ 1)	0.0810
MESO	1.02(0.99 ~ 1.05)	0.3100
OV	1(0.98 ~ 1.01)	0.6500
PAAD	0.97(0.94 ~ 1.01)	0.1100
PCPG	1.01(0.96 ~ 1.07)	0.6500
PRAD	1.14(1 ~ 1.31)	0.0530
READ	1.01(0.97 ~ 1.06)	0.5800
SARC	1(0.99 ~ 1.02)	0.4900
SKCM	1.01(1 ~ 1.02)	0.0520
STAD	0.99(0.97 ~ 1.02)	0.5200
TGCT	0.97(0.82 ~ 1.14)	0.7000
THCA	1.03(0.94 ~ 1.14)	0.5200
THYM	1.03(0.98 ~ 1.08)	0.2500
UCEC	1.02(1 ~ 1.05)	0.0200
UCS	0.99(0.96 ~ 1.01)	0.3400
UVM	1(0.92 ~ 1.08)	0.9400

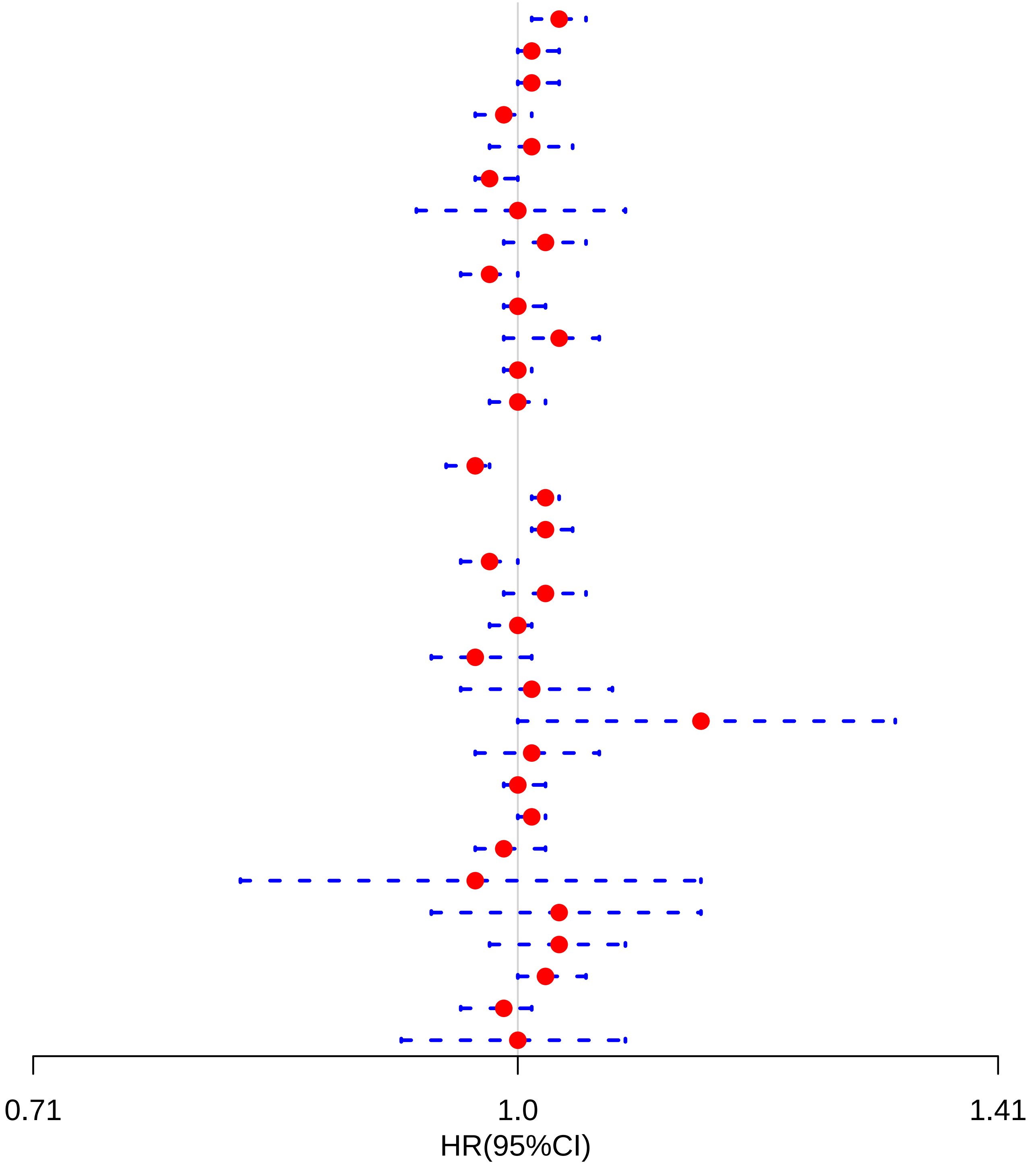

Supplement: Supplementary 4 — Gene expression of the CSID1 is significantly associated with disease-specific survival in cancers. [file 7823191.f4.pdf]
